# Low ketolytic enzyme levels in tumors predict ketogenic diet responses in cancer cell lines in vitro and in vivo[S]

**DOI:** 10.1194/jlr.M082040

**Published:** 2018-02-05

**Authors:** Jie Zhang, Ping-Ping Jia, Qing-Le Liu, Ming-Hua Cong, Yun Gao, Han-Ping Shi, Wei-Nan Yu, Ming-Yong Miao

**Affiliations:** Department of Endocrinology,* Huai’an Hospital Affiliated to Xuzhou Medical University, and Huai’an Second People’s Hospital, Huai’an 223002, Jiangsu, China; Department of Gastrointestinal Surgery/Clinical Nutrition,† Beijing Shijitan Hospital, Capital Medical University, Beijing 100038, China;; Department of Hyperbaric Oxygen Therapy,§ The First Affiliated Hospital of The Second Military Medical University, Changhai Hospital, Shanghai 200433, China; National Cancer Center/Cancer Hospital,** Chinese Academy of Medical Sciences and Peking Union Medical College, Beijing, 100021, China; Department of Biochemistry and Molecular Biology,†† The College of Basic Medical Sciences, The Second Military Medical University, Shanghai 200433, China

**Keywords:** ketogenic diet, ketone bodies, cancer, metabolism, BDH1, OXCT1, xenograft, HeLa, PANC-1

## Abstract

The ketogenic diet (KD) is a high-fat, very-low-carbohydrate diet that triggers a fasting state by decreasing glucose and increasing ketone bodies, such as β-hydroxybutyrate (βHB). In experimental models and clinical trials, the KD has shown anti-tumor effects, possibly by reducing energy supplies to cells, which damage the tumor microenvironment and inhibit tumor growth. Here, we determined expression levels of genes encoding the ketolytic enzymes 3-hydroxybutyrate dehydrogenase 1 (BDH1) and succinyl-CoA: 3-oxoacid CoA transferase 1 (OXCT1) in 33 human cancer cell lines. We then selected two representative lines, HeLa and PANC-1, for in vivo examination of KD sensitivity in tumors with high or low expression, respectively, of these two enzymes. In mice with HeLa xenografts, the KD increased tumor growth and mouse survival decreased, possibly because these tumors actively consumed ketone bodies as an energy source. Conversely, the KD significantly inhibited growth of PANC-1 xenograft tumors. βHB added to each cell culture significantly increased proliferation of HeLa cells, but not PANCI-1 cells. Downregulation of both BDH1 and OXCT1 rendered HeLa cells sensitive to the KD in vitro and in vivo. Tumors with low ketolytic enzyme expression may be unable to metabolize ketone bodies, thus predicting a better response to KD therapy.

Cancer is one of the most serious health problems in the world. The wide use of traditional cancer therapies, such as surgery, chemotherapy, or radiotherapy, is limited by indications, contraindications, side effects, and other considerations. The discovery of the Warburg effect provided a new direction for cancer treatment. Since then, cancer metabolism and nutrition have gradually become a hot topic of theoretical and clinical research (1). Cancer cells ferment glucose to lactate even in the presence of sufficient oxygen levels. This so-called aerobic glycolysis, a distinct characteristic of cancer cells, can be caused by gene mutations also found in mitochondrial disorders (2, 3). Thus, reducing carbohydrate supplementation may selectively cut off the energy supply of tumor cells, damage the tumor microenvironment, and thereby inhibit tumor growth (4).

The ketogenic diet (KD) is a high-fat, very-low-carbohydrate, and adequate protein diet that simulates a fasting metabolic state by decreasing glucose level while simultaneously elevating the levels of ketone bodies, β-hydroxybutyrate (βHB), and acetoacetate (AcAc). The initial clinical application of KD was to treat epilepsy (5). Numerous previous studies have indicated that this dietary restriction also reduces tumor volume and growth rate (6), improves patients’ quality of life (7), prolongs survival (8), and enhances the sensitivity to radiotherapy and chemotherapy (9, 10). In recent years, with more data becoming available on KD therapy and metabolic phenotypes of cancer cells, this diet has become popular among researchers and cancer patients due to its efficacy and potential anti-tumor effects. KD effects have also been shown to increase oxidative stress, suppress inflammation, enhance immune response, and affect various signaling pathways (11–14). However, in some studies, very different results have been reported when KD was examined as adjuvant therapy in mouse models (15–17).

In view of the completely opposite KD effects on cancer in different studies, the factors that influence the effectiveness of KD therapy need to be properly established. It is well known that ketone bodies are an important energy source during a KD. Whether cancer cells can utilize ketone bodies effectively is critically important to cell proliferation. Utilization of ketone bodies as energy source requires the presence of four enzymes: 3-hydroxybutyrate dehydrogenase 1/2 (BDH1/2), and succinyl-CoA:3-oxoacid CoA transferase 1/2 (OXCT1/2). Thus, we hypothesized that tumors with very low expression of these enzymes may have a better response to KD therapy. We investigated BDH1/2 and OXCT1/2 expression in several cancer cell lines and tested this hypothesis with two representative cell lines that had low and high expression levels of these key ketone body catabolism enzymes in vivo and in vitro.

## MATERIALS AND METHODS

### Cell lines and culture

All cell lines used in this study were purchased from Cell Bank, Chinese Academy of Sciences (Shanghai, China). Cells were cultured in DMEM or RPMI-1640 Medium with 10% FBS, 1% penicillin/streptomycin (Gibco, Grand Island, NY) and washed with PBS (Hyclone, Los Angeles, CA) every 48 h. All cells were cultured at 37°C in the atmosphere of 95% air and 5% carbon dioxide.

### Proliferation

For proliferation experiments, 5 × 10^4^ cells were planted per well in 6-well plates in DMEM or in low glucose (LG; 3 mM) medium with or without supplementation of 5 or 10 mM DL-β-hydroxybutyric acid sodium salt (Sigma-Aldrich, St Louis, MO). The medium with no βHB contained an equal volume of deionized water. βHB at the indicated concentration was added into the medium 5 h after first-day plating. Cell numbers were recorded at 12, 24, 48, 72, and 96 h by cell counting or using a Handheld Automated Cell Counter (Millipore, Billerica, MA). All wells were visually inspected to make sure that all cells were collected before counting.

### Lentivirus infection

*BDH1* and *OXCT1* siRNA target sequences and a scrambled control sequence were designed and cloned into iLenti siRNA vectors (ABM, Zhenjiang, China) that carried green fluorescent protein (GFP) and puromycin resistance genes by using convergent promoters U6 and H1. The RNA interference target sequences are shown in **Table 1** (four mixed target sequences for each). For lentivirus infection assay, cells were seeded on 6-well plates at a density of 2 × 10^5^/well. The next day, the cells were infected with lentivirus at a multiplicity of infection value of 10. GFP fluorescence signal was examined 72 h after the infection to ensure infection efficiency. In addition, 72 h after the infection, cells infected with the lentivirus were selected using 2.5 mg/ml of puromycin. quantative RT-PCR and Western blot analysiswere used to explore interferential efficiency.

**TABLE 1.** The RNA interference target sequences

Gene	Sequence(5′-3′)
BDH1	GCCCACTATTGCTTGGTTCTACTTCCTTT
	ACTCTGGATTTGGGTTCTCATTGGCCAAG
	AGCCTGAAGGACCCTGAGAAAGGCATGTG
	ACCTACAAGCAGGTGGCAGAAGTGAACCT
OXCT1	CCAGAGAATCTTATAGATGCTTTACTGAA
	TGCCATTGCCAGTAAGCCAAGAGAGGTGA
	TTGGAGCATTTGCTCCAGAAGACATCCAT
	TACCATTGACTGGAAAGCAATGTGTCAAC
Scramble	GGGTGAACTCACGTCAGAA

### RNA extraction and qRT-PCR

Total RNA was extracted using TRIzol Reagent (Invitrogen, Carlsbad, CA) following manufacturer’s instructions. CDNA was synthesized following the manufacturer’s protocol. qRT-PCR was performed by applying Takara SYBR Premix Ex Taq II (Bio-Rad, Hercules, CA). Gene-specific PCR amplification was performed using an ABI 7300 instrument (Applied Biosystems, Darmstadt, Germany). The primers listed in **Table 2** were synthesized by Sangon Biotech Co. (Shanghai, China). All genes were amplified in the same reaction as internal loading control. Amplification specificity was confirmed by melting curves; the fluorescence was determined at 60°C. PCR conditions were as follows: predegeneration at 95°C for 30 s, followed by 40 cycles for 5 s at 95°C and 31 s at 60°C. qRT-PCR reactions were performed in a total volume of 20 µL. All experiments were performed in triplicate. Relative quantitative expression levels were calculated by using the ΔCt^−1^ or 2^-ΔΔCt^ method.

**TABLE 2.** qRT-PCR primer sequences

Gene	Orientation	Sequences(5′-3′)
BDH1	Forward	GAAAGTGGTGGAGATTGTCCGC
	Reverse	TGTAGGTCTCCAGGCTGGTGAA
BDH2	Forward	TTCCAGCGTCAAAGGAGTTGT
	Reverse	TTCCTGGGCACACACAGTTG
OXCT1	Forward	GCTTTGGTGAAAGCCTGGAAGG
	Reverse	CTCTACCACTGTGGTTTCTGCAG
OXCT2	Forward	AACGGCGACCACTTCCTTTT
	Reverse	ACATGGGCACGTTGAAATTGC
β-actin	Forward	CCTGGCACCCAGCACAAT
	Reverse	GGGCCGGACTCGTCATAC

### Western blot analysis

To extract total protein, cells were washed twice with cold PBS and lysed in 1 ml RIPA buffer (50 mM Tris,150 mM NaCl,1% NP-40,0.5% sodium deoxycholate) and debris was removed by centrifugation at 13,500 *g* at 4°C. Polyvinylidene fluoride membranes (Millipore) were incubated with specific antibodies against BDH1, OXCT1 (at dilutions of 1:500 and 1:1000, respectively; Proteintech, Chicago, IL) and β-actin (Sangon). Then, samples were incubated with HRP-coupled anti-mouse secondary antibodies (Sangon) and visualized using enhanced chemiluminescence (Pierce, Rockford, IL).

### Immunohistochemical staining

Xenograft tumor tissue samples were fixed in 10% formalin, embedded in paraffin and cut into 4 μm-thick sections by using routine methods. For immunohistochemical staining, all procedures were performed according to the manufacturer’s protocol. The BDH1 and OXCT1 antibodies (Proteintech) were diluted at 1:50 and 1:200, respectively. Color development was carried out using chromogen (3, 3′-diaminobenzidine) reagent and hematoxylin was used as a counterstain. Finally, the slides were examined using a light microscope.

### Animal models

All experimentation on animals was approved by the Institutional Animal Care and Use Committee at the Second Military Medical University. For tumor implantation, nude mice (nu/nu, male, aged 4 weeks, SLEC, Shanghai, China) were housed in a specific pathogen-free facility. One week later, a suspension of 2 × 10^6^ HeLa cells in 200 μl PBS or of 1 × 10^7^ PANC-1 cells in 300 μl PBS was inoculated subcutaneously into the lateral aspect of the rear leg. Tumor growth was recorded every 3 days starting from 2 weeks after inoculation by measuring two perpendicular diameters using the following formula: π/6 × length × width^2^.Then the nude mice were randomly distributed into two groups: a standard diet (STD) group and a ketogenic diet (KD) group. KD was given at a dose of 125 g/kg (Zeneca, Shenzhen, China), which was based on three preexperiments. Thus, the calorie intake of the KD-fed group was almost equal to the STD-fed group. Average food consumption and the main composition of the diets are shown in supplementary Table S1.Tumor volume was measured for 4 weeks using electronic calipers. Mouse body weight was monitored during the experiments. Mouse blood was obtained from tail clip and blood glucose and βHB levels were measured using the Freestyle Optium Blood Glucose and Ketone Monitoring System (Abbott Diabetes Care Ltd., Oxford, UK). We also established other paired animal models, as described above, to explore mean survival. The terminal criteria for the transplanted mice was as follows: every mouse suffering from any obvious discomfort, impending death, systemic signs of unhealth, or any condition that was likely a harbinger of impending discomfort or death would be euthanized.

### Statistical analysis

SPSS 19.0 and GraphPad Prism 7.0 were used for statistical analysis. The Kaplan-Meier method and log-rank test were applied to determine the difference in mouse survival. Differences in tumor volume, body weight, blood glucose, blood βHB, and gene expression levels in xenograft tumors were evaluated by the two-tailed Student’s *t*-test in STD and KD groups. Cell proliferation was analyzed by one-way ANOVA followed by the least significant difference multiple comparison post hoc test when appro­priate. All statistical analyses were conducted with a significance level of α = 0.05 (*P* < 0.05).

## RESULTS

### Expression of key ketolytic enzymes BDH1/2 and OXCT1/2 in 33 cancer cell lines

**Fig. 1.**
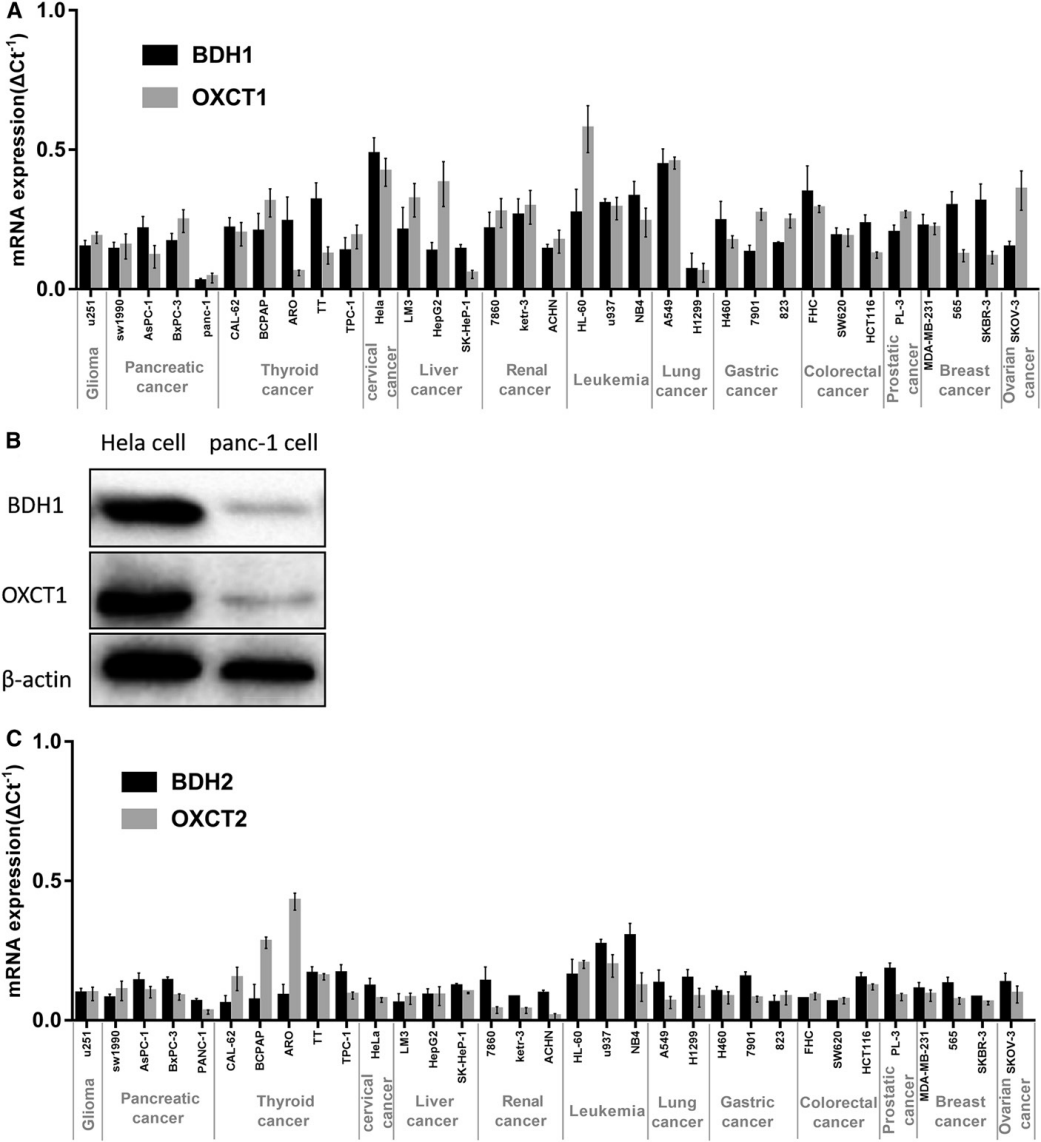
Expression of key ketolytic enzymes BDH1 and OXCT1 in 33 cancer cell lines. A: BDH1 and OXCT1 mRNA expression levels in 33 tumor cell lines. Relative quantitative expression levels were calculated by using the ΔCt ^-1^ method. B: BDH1 and OXCT1 protein expression levels in PANC-1 cells and HeLa cells. C: BDH2 and OXCT2 mRNA expression levels in 33 tumor cell lines.

Expression levels of *BDH1/2* and *OXCT1/2* mRNAs in 33 cancer cell lines were determined by qRT-PCR. As shown in **Fig. 1**, these key ketolytic enzymes were expressed in all cell lines, but their expression levels significantly varied in different lines. However, BDH1 and OXCT1 may play a more dominant role than BDH2 and OXCT2. For further experiments, we chose PANC-1 and HeLa cells with low and high expression levels of both *BDH1* and *OXCT1* mRNAs, respectively. We also detected the expression of the BDH1 and OXCT1 proteins in PANC-1 and HeLa cells by Western blotting analysis. The protein levels were consistent with mRNA expression levels (Fig. 1B).

During this study, we hypothesized that cancers with high glycometabolism (which means enhanced glycolysis in cancer cells here) may be characterized by a lower level of ketolytic key enzymes. Thus, we examined relevant glycometabolic markers: glucose transporter 1 (GLUT1), glucose transporter 3 (GLUT3), transketolase like-1 (TKTL-1). However, no correlation between glycometabolism and ketone body metabolism was observed (supplementary Fig. S1).

### Lack of effect of βHB-supplemented LG medium on the proliferation of PANC-1 cells

**Fig. 2.**
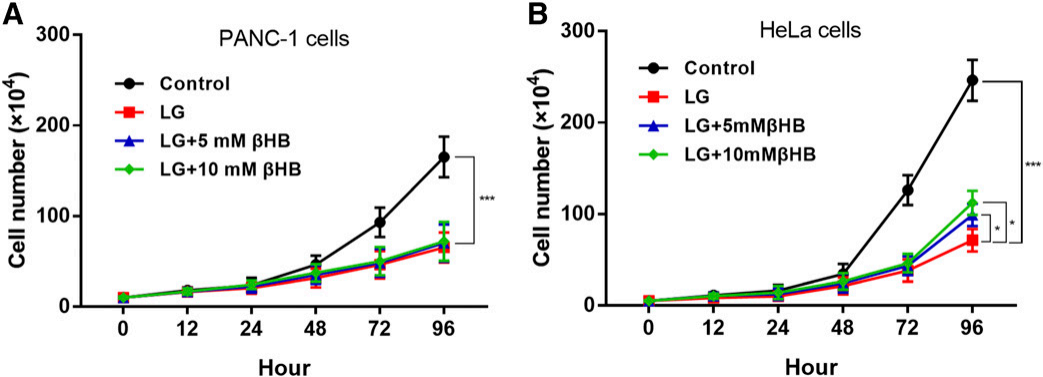
Lack of effect of βHB-supplemented LG medium on the proliferation of PANC-1 cells. A: Proliferation of PANC-1 cells was significantly inhibited in LG medium compared with that in control medium (***P < 0.001). In the presence of βHB in LG conditions, the lower rate of proliferation of PANC-1 cells was not increased at any time point. B: Proliferation of HeLa cells was significantly inhibited in LG medium compared with that in control medium (***P < 0.0001). Proliferation of HeLa cells cultured for 72 h in βHB-supplemented LG medium was significantly higher than that of cells cultured in LG media alone (*P < 0.05 and ***P < 0.001 for 5 mM and 10 mM βHB, respectively)

Next, we found that the proliferation of PANC-1 and HeLa cells was significantly decreased during culturing in LG medium for 96 h compared with that in control, high glucose (25 mM) medium (*P* < 0.0001, **Fig. 2**). In the presence of 5 mM or 10 mM βHB in LG conditions, a lower rate of proliferation of PANC-1 cells was not affected at any time point (Fig. 2A). In contrast, proliferation of HeLa cells cultured for 72 h in βHB-supplemented LG medium was significantly higher than that of cells cultured in LG medium alone (*P* < 0.05 and *P* < 0.0001 for 5 mM and 10 mM βHB, respectively; Fig. 2B). Meanwhile, we also added 10 mM βHB into the control medium, which had no significant influence on the proliferation of HeLa cells compared with control medium (supplementary Fig. S2).

### BDH1 and OXCT1 knockdown in HeLa cells by lentivirus-mediated RNA interference

**Fig. 3.**
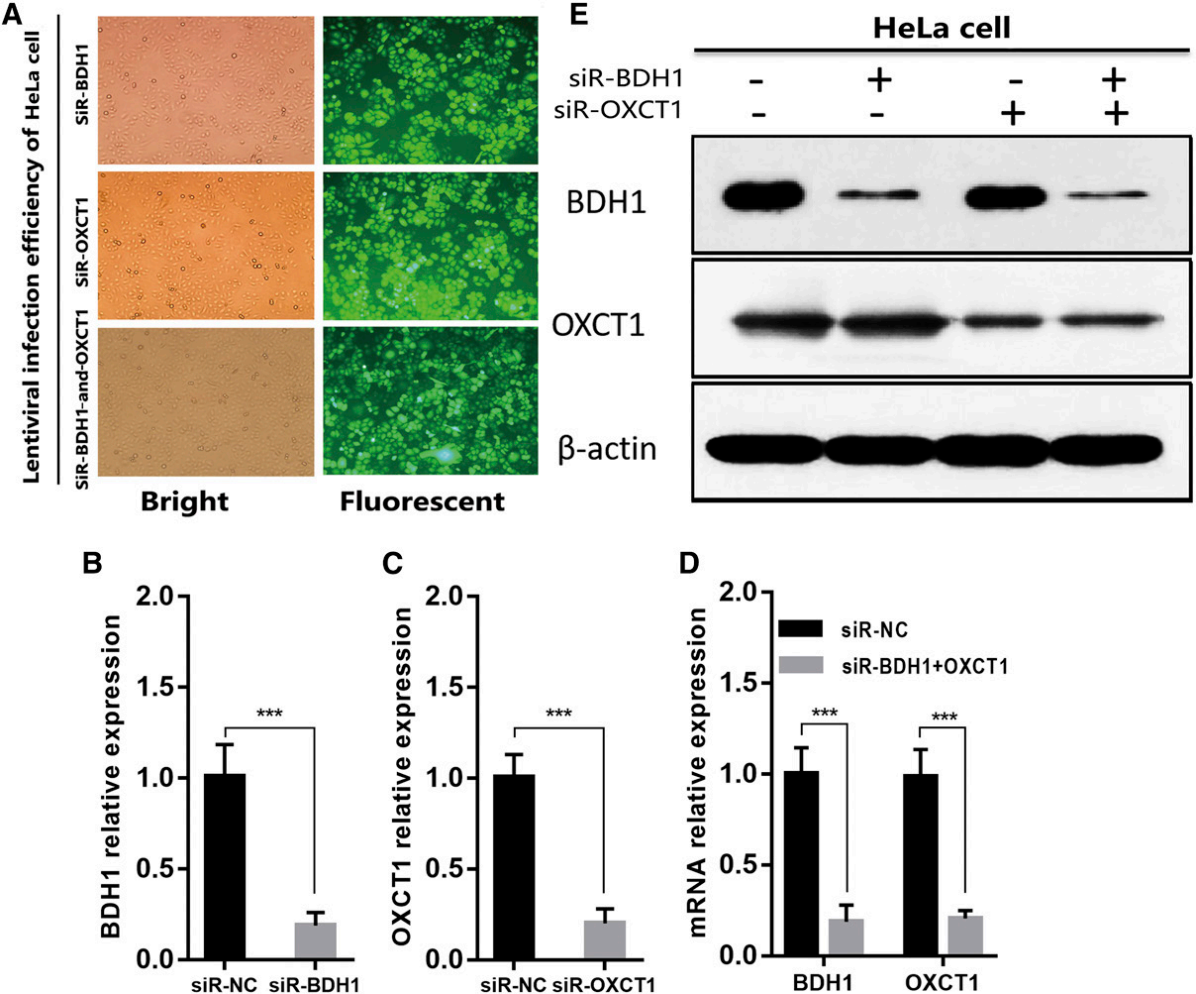
BDH1 and OXCT1 knockdown in HeLa cells by lentivirus-mediated RNA interference. A: Representative images of Hela cells infected with lentivirus expressing either siR-NC, siR-BDH1, or siR-OXCT1 after lentivirus infection and selected by puromycin. B–D: *BDH1* and *OXCT1* expressions were analyzed by qRT-PCR, and their expressions were downregulated obviously at the RNA levels in HeLa cells through lentivirus-mediated RNA interference. β-actin was used as an internal control, and the data represent the mean ± SD of three independent experiments (***P < 0.001). E: BDH1 and OXCT1 protein expression levels were inhibited significantly in HeLa cells through lentivirus-mediated RNA interference.β-actin was used as an internal control.

In the next series of experiments, by using lentivirus-mediated RNA interference, we knocked down BDH1 and OXCT1 separately or simultaneously in HeLa cells. The highest infection efficiency was obtained at a multiplicity of infection value of 10 as more than 90% of HeLa cells showed GFP signal after lentivirus infection and selection by puromycin (**Fig. 3A**). Gene knockdown efficiency was assessed by qRT-PCR and Western blotting. *BDH1* and *OXCT1* mRNA expression levels were reduced to approximately 20% of those in control cells (Fig. 3B–D). BDH1 and OXCT1 protein levels were also significantly suppressed (Fig. 3E). These findings confirmed that BDH1 and OXCT1 expression levels in HeLa cells were successfully knocked down by lentivirus infection. As a result of these experiments, we obtained siR-NC (expressing scrambled nonsilencing siRNA), siR-BDH1, siR-OXCT1, and siRNA-BDH1-and-OXCT1 HeLa cells.

### Supplementation of LG medium with βHB does not affect proliferation of HeLa cells with downregulated BDH1 and OXCT1

**Fig. 4.**
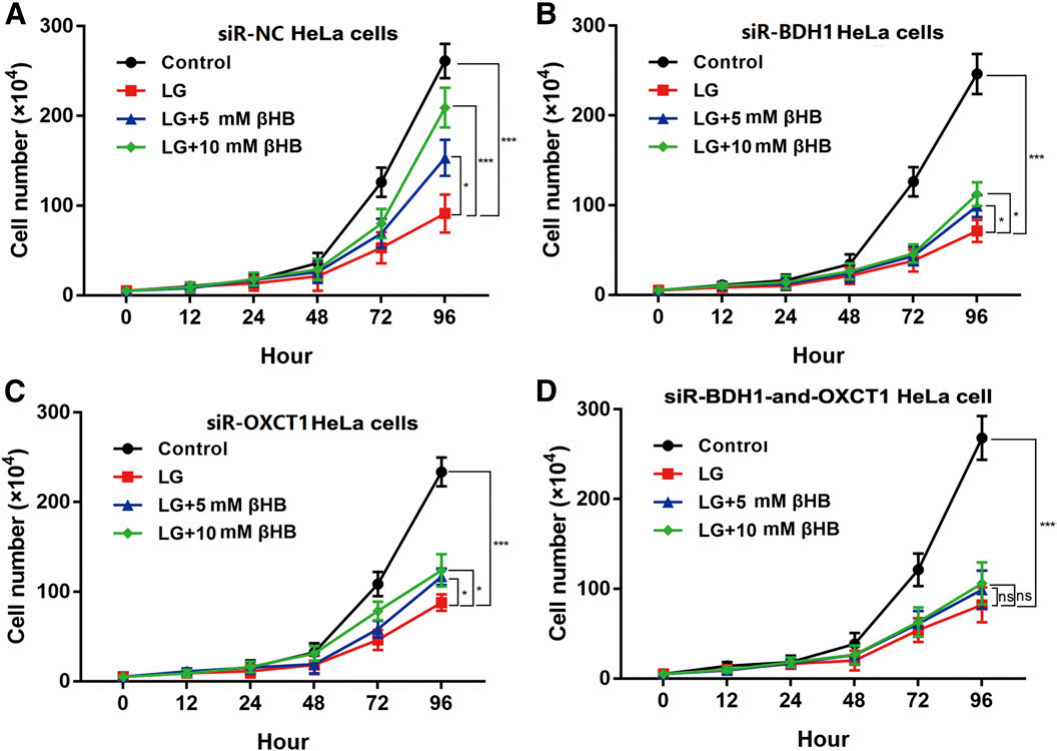
Supplementation of LG medium with βHB does not affect proliferation of HeLa cells with downregulated BDH1 and OXCT1. A: Proliferation of siR-NC HeLa cells cultured for 72 h in βHB-supplemented LG medium was significantly higher than that of cells cultured in LG medium alone (*P < 0.05 and ***P < 0.001 for 5 mM and 10 mM βHB, respectively. B, C: Proliferation of siR-BDH1 and siR-OXCT1 HeLa cells was increased in the presence of 5 mM or 10 mM βHB in LG medium (*P < 0.05). D: Proliferation of siRNA-BDH1-and-OXCT1 HeLa cells was not affected by βHB supplementation in LG medium with βHB. Values are indicated as mean ± SD of three separate experiments.

After incubation with lentiviruses, the four groups of HeLa cells were cultured and subjected to cell proliferation assays. As shown in **Fig. 4A**, the proliferation curve of siR-NC HeLa cells in different conditions, with or without βHB supplement, was similar to that of HeLa cells that were not infected with lentivirus (Fig. 2A). As shown in Fig. 4B and C, proliferation of siR-BDH1 and siR-OXCT1 HeLa cells was increased in the presence of 5 mM or 10 mM βHB in LG medium (*P* < 0.05). Remarkably, however, proliferation of siRNA-BDH1-and-OXCT1 HeLa cells was not affected by βHB supplementation and remained similar to that in LG medium without βHB (Fig. 4D).

### KD inhibited growth of PANC-1 but not of HeLa cell xenograft tumors in nude mice

**Fig. 5.**
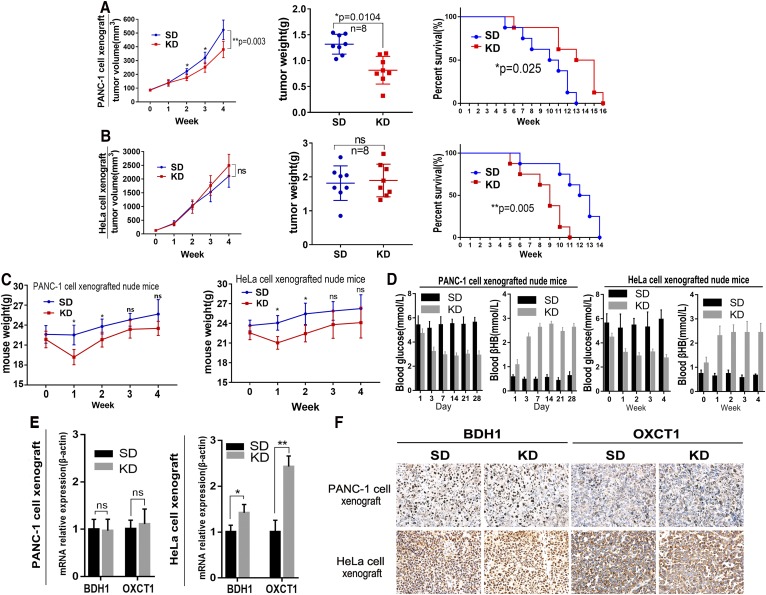
KD inhibited growth of PANC-1 but not HeLa cell xenograft tumors in nude mice. A: The KD was associated with significantly lower growth and weight of PANC-1 xenograft tumors in comparison to the same parameters in the STD group (*P < 0.05, left and middle panels). The KD also significantly prolonged the mean survival of mice with PANC-1 xenograft tumors (*P < 0.05, right panel). Kaplan-Meier method and log-rank test were applied to determine mouse survival. B: KD nominally increased tumor growth (left) and weight (middle) in HeLa cell-xenografted nude mice and significantly decreased the mean survival (**P < 0.01). C: Body weight of the xenografted mice. Data are expressed as mean ± SD. *P < 0.05 compared with the control. D: The blood glucose and βHB levels in PANC-1 and HeLa xenografted mice. (E, F) The expressions of BDH1 and OXCT1 mRNA and protein in xenografted tumors. Data are expressed as mean ± SD; n = 3; *P* values were obtained by a two-tailed Student’s *t*-test. For protein expression, positive expression was shown as yellow or brown.

We next examined whether the use of KD in vivo led to results consistent with the in vitro data presented above. We found that KD was associated with significantly lower growth and weight of PANC-1 xenograft tumors in comparison to the same parameters in the STD group (*P* < 0.05, **Fig. 5A**, left and middle panels). Moreover, KD significantly prolonged the mean survival of mice with PANC-1 xenograft tumors (*P* < 0.05, Fig. 5A, right panel). In contrast, KD was associated with nominally higher tumor growth rate and tumor weight in mice with HeLa xenograft tumors but the effect did not reach statistical significance (Fig. 5B, left and middle panels). Furthermore, the mean survival of mice with HeLa xenograft tumors that received a KD was significantly lower than that of mice in STD group (*P* < 0.05; Fig. 5B, right panel).

In addition, we measured mouse body weight weekly. We found that the mean body weight of KD-fed mice was significantly lower than that of STD-fed mice during the first 2 weeks (*P* < 0.05; Fig. 5C). However, after 2 weeks, this difference between the two groups was not significant. KD also lowered blood glucose and elevated blood βHB significantly (*P* < 0.05; Fig. 5D). Finally, we detected BDH1 and OXCT1 expression levels in tumor tissue by qRT-PCR and immunohistochemistry. As shown in Fig. 5E and F, there was no increased expression of BDH1 and OXCT1 mRNA and protein in PANC-1 xenograft tumors in the KD group compared with their levels in mice from the STD group. However, in mice with HeLa cell xenograft tumors, KD was associated with significantly higher BDH1 and OXCT1 expression levels as demonstrated by qRT-PCR (*P* < 0.05 and *P* < 0.01, respectively), and immunohistochemical staining also showed a significant difference in staining intensity for these proteins between the two groups. We also examined H1229 lung cancer cells with low expression of BDH1 and OXCT1. KD did not inhibit growth of tumors derived from H1299 cell xenografts in nude mice. We reexamined the expression of BDH1 and OXCT1 in tumor tissues and found that these two genes were significantly upregulated (supplementary Fig. S3).

Furthermore, we compared the expression levels of BDH1 and OXCT1 in PANC-1, H1299, and HeLa cell xenografts in the KD group.The BDH1 and OXCT1 expression levels in H1299 cell xenografts were lower than the ones in HeLa cells (*P*<0.05), but significantly higher than the ones in PANC-1 xenografts (*P*<0.01, supplementary Fig. S4).

### KD inhibited growth of siR-BDH1-and-OXCT1 HeLa cell xenograft tumors in nude mice

**Fig. 6.**
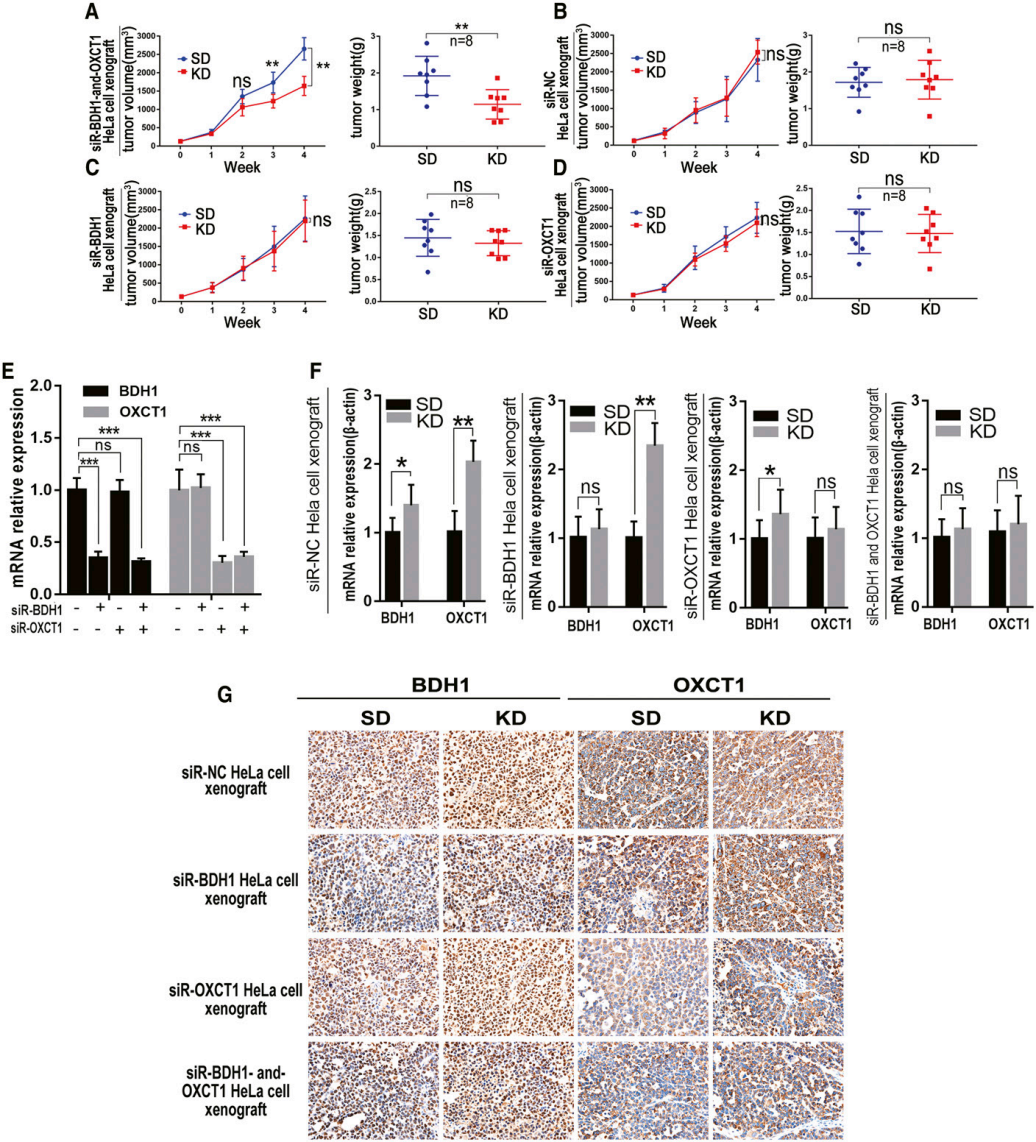
KD inhibited growth of siR-BDH1-and-OXCT1 HeLa cell xenograft tumors in nude mice. A–D: Tumor growth (left) and weight (right) of xenografted nude mice. Data are expressed as mean ± SD. *P < 0.05, **P < 0.01, ***P < 0.001 compared with the control. E: The *BDH1* and *OXCT1* mRNA expression levels in xenograft tumors in STD-fed mice. The relative quantitative expression was calculated by the 2^-ΔΔCt^ method. *P < 0.05, **P < 0.01, ***P < 0.001 compared with the control. F: The *BDH1* and *OXCT1* mRNA expression levels in xenograft tumors by qRT-PCR. Relative quantitative expression was calculated by the 2^-ΔΔCt^ method. *P < 0.05, **P < 0.01, ***P < 0.001 compared with the control. G: The BDH1 and OXCT1 protein expression levels in xenograft tumors by immunohistochemical staining. Positive expression was shown as yellow or brown.

We also observed that KD treatment suppressed tumor growth in nude mice with xenografts derived from HeLa cells with downregulated BDH1 and OXCT1. The difference in mean tumor volume between mice with siR-BDH1-and-OXCT1 HeLa cell xenografts in the KD and STD groups at the third week was statistically significant (*P* < 0.01; **Fig. 6A**). However, KD did not significantly inhibit tumor growth rates of xenografts derived from siR-NC, siR-BDH, or siR-OXCT1 HeLa cells (Fig. 6B–D, respectively). Mouse weight, blood glucose, blood βHB, and mean survival time were all similar to those in nude mice with HeLa cell xenografts as above (data not shown). We also detected expression levels of BDH1 and OXCT1 mRNA and protein and observed the inhibitory effect of RNA interference in the xenografts (Fig. 6E, F).

## DISCUSSION

In the present study, we investigated the relationship between the expression of the key ketolytic enzymes BDH1 and OXCT1 in tumors and their response to KD. We showed that xenograft tumors that derive from cancer cells with low expression levels of BDH1 and OXCT1 are more responsive to KD therapy, likely because they possess weaker ability to metabolize ketone bodies.

The Warburg effect is one of the most remarkable and ubiquitous metabolic phenotypes exhibited by most cancer cells (18). Exploiting metabolic characteristics of tumors and using targeted therapeutic strategy may prove effective against cancers of the same category. Theoretically, cancer metabolic deficiency caused by gene mutations or mitochondrial dysfunction can prevent cancer cells from effectively consuming ketone bodies as a source of energy (19). If a tumor is unable to utilize ketone bodies, the use of KD may be an effective therapy for selective nutrient starvation of such tumors. However, numerous reports about inconsistent efficacies of this treatment broke the illusion of the possibility to cure cancer thoroughly with KD therapy.

In our study, supplementing LG medium with βHB did not promote PANC-1 cell growth in vitro (Fig. 2A), whereas proliferation of HeLa cells was significantly increased by βHB (Fig. 2B). Similar results were observed in xenograft experiments in nude mice (Fig. 5A, B). Notably, HeLa cells apparently could not consume ketone bodies as fuel to promote proliferation when BDH1 and OXCT1, the main ketolytic enzymes, were knocked down simultaneously (Fig. 6A).

**Fig. 7.**
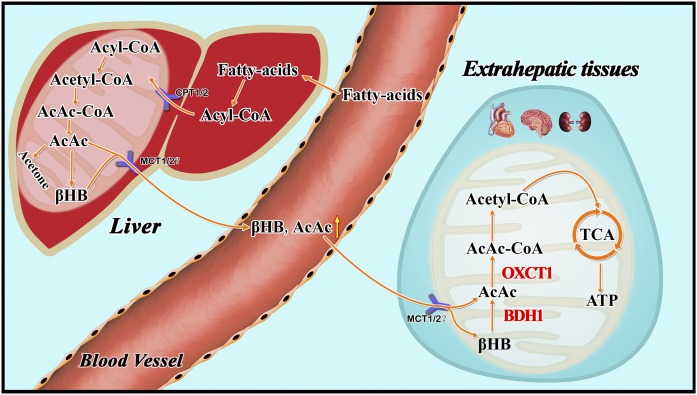
Metabolism of ketone bodies. Ketogenesis occurs in the hepatic mitochondrial matrix.Then AcAc and βHB are released to extrahepatic tissues via monocarboxylate transporters MCT1 and MCT2, mainly.The AcAc/βHB ratio is proportional to the mitochondrial NAD^+^/NADH ratio (often ∼30% to 70%). BDH1 and OXCT1 are two ketolytic key enzymes. In the extrahepatic mitochondrion, BDH1 catalyzes βHB converting to AcAc and then AcAc is transformed into AcAc-CoA in a near-equilibrium reaction catalyzed by OXCT1.

However, previous studies have shown ketone bodies (βHB and AcAc) may be much more than metabolites, having important cellular signaling roles as well (20, 21). It was unknown whether βHB promoted the proliferation of HeLa cells through some signaling pathways independent of its role in cell metabolism. Supplementation of control (high glucose) medium with 10 mM βHB did not affect proliferation of HeLa cells. This result indicates that the main role of βHB in HeLa cells is to serve as an energy source. Our results suggest that cancer cells with very low expression levels of BDH1 and OXCT1 may be sensitive to KD therapy. In relevance to this notion, in a recent clinical trial, Chang et al. (22) found differential expression of ketolytic enzymes (including BDH1 and OXCT1) in gliomas. They hypothesized that patients with low or very low expression of BDH1 and OXCT1 in malignant gliomas may respond better to KD therapy. Ketogenesis occurs in hepatic mitochondrial matrix (**Fig. 7**). AcAc and βHB are released to extrahepatic tissues mainly via monocarboxylate transporters MCT1 and MCT2 (23). The AcAc:βHB ratio is proportional to the mitochondrial NAD^+^:NADH ratio (often ∼30–70%). Thus, in an extrahepatic mitochondrion, BDH1 catalyses oxidation of βHB into AcAc, and then AcAc is transformed into AcAc-CoA in a near-equilibrium reaction catalyzed by OXCT1. Extraneous AcAc can be also converted into AcAc-CoA directly. In vitro, siR-BDH1 or siR-OXCT1 HeLa cells had limited proliferation in LG medium supplemented with βHB, which suggested that they partly retained the ability to consume βHB as energy source. However, KD did not inhibit growth of xenograft tumors derived from siR-BDH1 or siR-OXCT1 HeLa cells in nude mice. Due to a complex internal environment, we have not found a reasonable explanation for the observed phenomenon. We believe that these observations point to some unknown mechanism of ketone body metabolism.

We also determined the levels of BDH2 and OXCT2 that play a certain role in ketone body catabolism. BDH2 and OXCT2 levels in most cell lines were lower than those of BDH1 and OXCT1 (Fig. 1). Previous studies have shown that BDH2 catalyzes a rate-limiting step in the biogenesis of the mammalian siderophore and OXCT2 is a testis-specific isoform mostly expressed in testicular haploid germ cells (24, 25). Thus, during catabolism of the ketone body, BDH1 and OXCT1 may play a more dominant role than the isoforms BDH2 and OXCT2.

Cancer cells reactivate gene expression or metabolic pathways to achieve proliferation under nutrition-limiting conditions (26, 27). In our study, we determined BDH1 and OXCT1 expression levels in transplanted tumors. Surprisingly, we did not observe increased expression of BDH1 and OXCT1 in tumors derived from PANC-1 cell xenografts. The lack of elevated expression of these two key ketone catabolism enzymes suggests that ketolysis was not activated in xenograft tumors. In contrast, qRT-PCR showed upregulated levels of *BDH1* and *OXCT1* in HeLa cell xenografts. Metabolic adaptations, such as reactivation of key ketone catabolism enzymes, may be one of the main reasons for failure of KD therapy on lung cancer cell xenografts.This metabolic adaptation can be also observed in nutrition-deprived hepatocellular carcinoma cells that employ ketone bodies for energy supply and cancer progress (28). Therefore, investigating the potential mechanism by which gene expression is reactivated may further enhance anti-tumor effects of KD therapy.

In this study, we also observed that KD elevated blood βHB, decreased blood glucose, and lowered mouse body weight. (Fig. 5D). KD-fed mice had significantly higher ketone body concentrations, a feature which was also observed in other studies using a KD in tumor bearing mice (29). It may underlie the efficacy of KD therapy against cancer. These mice lost 10–20% of their body weight compared with STD-fed animals during the first week. Although the mean mouse body weight in the KD group was lower than that in the STD group at every time point, the difference during the last week was not statistically significant. These results were consistent with the data of several previous studies (30, 31). To avoid weight loss, KD was administered unlimitedly or with high energy content in most studies (7, 32). However, different nutrition ratios may significantly influence the efficacy of KD. In this study, we employed KD with the lipid:nonlipid ratio of 3:1. In fact, 4:1 or 5:1 KD may have an even better effect than a 3:1 diet. However, animals do not always tolerate the higher fat proportion well (33). In addition, the KD even increased tumor growth and shortened the mean survival mice xenografted with HeLa cells. This finding suggests that HeLa cell-derived xenograft tumors actively consume ketone bodies as an energy source which led to significant growth of tumor tissue and energy deficit in normal tissue.

Nevertheless, overall, our results suggest that expression of the key ketolytic enzymes BDH1 and OXCT1 is one of the main factors that determine the effect of a KD on cancer. Tumors with low expression of BDH1 and OXCT1 may respond better to KD therapy than those with high expression of these proteins. BDH1 and OXCT1 may be very useful biomarkers to determine the sensitivity of a particular cancer to a KD. The use of BDH1 and OXCT1 inhibitors may enhance KD treatment efficiency and expand the anti-tumor spectrum of KD therapy. Further studies are necessary to determine whether other tumors derived from cancer cells with different expression levels of BDH1 and OXCT1 exhibit similar sensitivity to KD. Furthermore, the mechanism by which KD therapy activates BDH1 and OXCT1 expression should be elucidated. Finally, it is necessary to determine whether other sensitive indicators can also affect KD effectiveness.

## Supplementary Material

Supplemental Data
