# Supplementary material for: Low ketolytic enzyme levels in tumors predict ketogenic diet responses in cancer cell lines in vitro and in vivo
Source: J Lipid Res. 2018 Feb 5;59(4):625–34. doi: 10.1194/jlr.M082040 (PMC5880499; doi:10.1194/jlr.M082040)
Supplement: Supplemental Data [file supp_59_4_625__index.html]

Low ketolytic enzyme levels in tumors predict ketogenic diet responses in cancer cell lines in vitro and in vivo — Low ketolytic enzyme levels in tumors predict ketogenic diet responses in cancer cell lines in vitro and in vivo — Supplemental Data 

# Low ketolytic enzyme levels in tumors predict ketogenic diet responses in cancer cell lines in vitro and in vivo

## Supplemental Data

- supplemental information (.pdf, 783 KB) - supplemental information
