# Supplementary material for: Low ketolytic enzyme levels in tumors predict ketogenic diet responses in cancer cell lines in vitro and in vivo
Source: J Lipid Res. 2018 Feb 5;59(4):625–34. doi: 10.1194/jlr.M082040 (PMC5880499; doi:10.1194/jlr.M082040)
Supplement: Supplemental Data [file 10.1194_M082040_jlr.M082040-1.pdf]

SUPPLEMENTAL INFORMATION:

Low ketolytic enzyme levels in tumors predict ketogenic diet responses in cancer cell lines in vitro and in vivo

Jie Zhang[1, 5#], Ping-Ping Jia[2#], Qing-Le Liu[3#], Ming-Hua Cong[4], Yun Gao[5], Han-Ping Shi[2]*, Wei-Nan Yu[1]*, Ming-Yong Miao[5]*

[1]Department of Endocrinology, Huai'an Hospital Affiliated to Xuzhou Medical University, and Huai'an Second People's Hospital, Huai'an 223002, Jiangsu, China. [2]Department of Gastrointestinal Surgery / Clinical Nutrition, Beijing Shijitan Hospital, Capital Medical University, Beijing 100038, China. [3]Department of Hyperbaric Oxygen Therapy, The First Affiliated Hospital of The Second Military Medical University, Changhai Hospital, Shanghai 200433, China. [4]Department of Comprehensive Therapy, Cancer Hospital of Chinese Academy of Medical Sciences, Beijing 100021. [5]Department of Biochemistry and Molecular Biology, The College of Basic Medical Sciences, The Second Military Medical University, Shanghai 200433, China.

Table S1. The exact composition of the diets

	Standard diet (%)	Ketogenic diet (%)
**Protein**	19.0	18.2
**Fat**	4.0	62.8
**Carbohydrate**	50.0	2.6
**Fiber**	5.0	4.8
**Ash content**	⩽8.0	5.2
**Water content**	<10.0	<10.0
**others**	vitamins[1] and trace elements	
**Calories per gram**	6.49kcal	3.12kcal

Note: [1]The exact composition of vitamins are shown in table S2.

Table S2. The exact composition of the vitamins

	Value	Unit
Vitamin A	13800	1U/kg
Vitamin B12	19	mcg/kg
Ascorbic Acid	0	mg /kg
Vitamin D3	1900	1U/kg
Vitamin E	220	1U/kg
Vitamin K3	2	mg/kg
Biotin	0.38	mg /kg
Choline	1790	mg /kg
Folic acid	3.8	mg/kg
nicotinic acid	57.1	mg /kg
Pantothenic acid	28	mg /kg
Pyridoxine	11	mg /kg
Rinoflavin	11.5	mg /kg
Thiamine	11.4	mg/kg

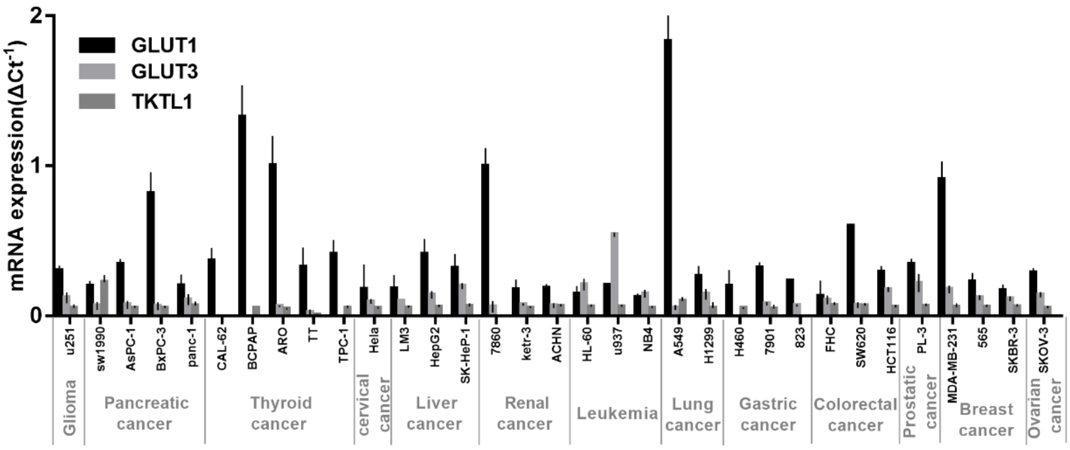

Fig. S1. The mRNA expression levels of relevant glycometabolic markers in 33 cancer cells by qRT-PCR.

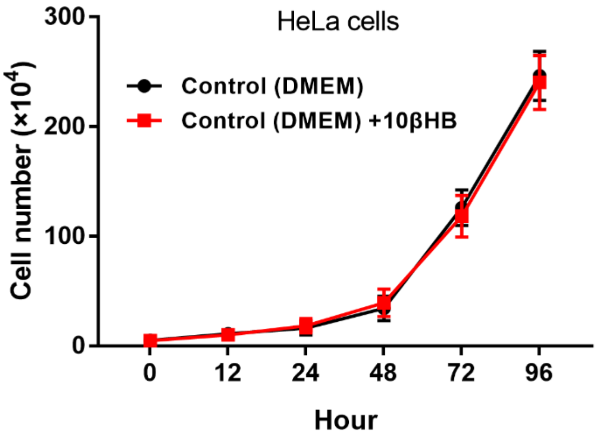

Fig. S2. Supplementation of control medium with 10 mMβHB does not affect proliferation of HeLa cells.

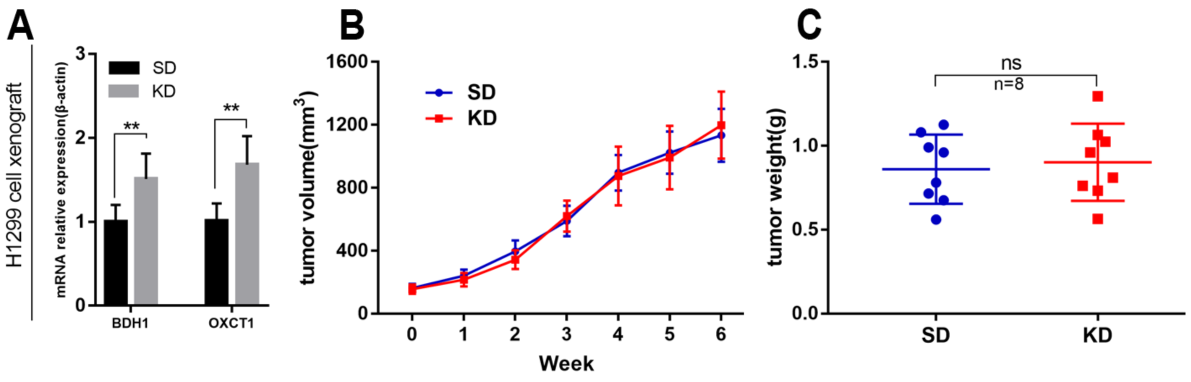

Fig. S3. KD could not inhibit tumor growth in H1299 cell xenografted nude mice. (A) The expression levels of BDH1 and OXCT1 mRNA in xenograft tumors by qRT-PCR. The relative quantitative expression was calculated by the $2^{-\Delta\Delta Ct}$ method. **p<0.01 compared with the control. **(B)** Tumor growth of xenografted nude mice. **(C)** Tumor weight of xenografted nude mice.

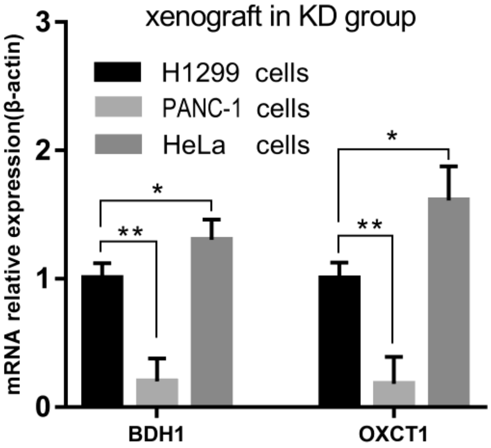

Fig. S4. The expression levels of BDH1 and OXCT1 in PANC-1, H1299 and HeLa cell xenografts in KD group. The BDH1 and OXCT1 expression levels in H1299 cell xenograft were lower than the ones in HeLa cell (*P<0.05), but significantly higher than the ones in PANC-1 xenograft (**P<0.01).
